# Clinical outcomes following the surgery of new autologous arteriovenous fistulas proximal to the failed ones in end-stage renal disease patients: a retrospective cohort study

**DOI:** 10.1080/0886022X.2019.1696210

**Published:** 2019-12-09

**Authors:** Xianglei Kong, Lijun Tang, Liming Liang, Wei Cao, Lei Zhang, Wei Yong, Nannan Ding, Wenbin Li, Zunsong Wang, Dongmei Xu

**Affiliations:** a Department of Nephrology, Shandong Provincial Qianfoshan Hospital, Shandong University, Jinan, China;; b Department of Nephrology, The First Affiliated Hospital of Shandong First Medical University, Jinan, China;; c Shandong Provincial Key Laboratory for Rheumatic Disease and Translational Medicine, Jinan, China;; d Nephrology Research Institute of Shandong Province, Jinan, China

**Keywords:** Autologous arteriovenous fistula, surgery, prognosis, mortality

## Abstract

**Background:** Most prior studies have explored surgery for the treatment of failed autologous arteriovenous fistulas (AVFs) with limited follow-up times and a lack of end point mortality. Accordingly, we conducted a retrospective cohort study to evaluate the clinical outcomes of the surgery of new AVF proximal to the failed forearm AVF.

**Methods**: In this study, 538 end-stage renal disease patients (group A, 418 with primary AVF; and group B, 120 with failed AVF) were consecutively enrolled between January 2013 and June 2016, with a median follow-up time of 41 months. Primary and secondary patency, all-cause mortality, and risk factors associated with AVF failure were explored by the Kaplan–Meier method or Cox proportional hazards model.

**Results**: In group A (*n* = 418), the primary and secondary patencies of AVF were 85.6% vs. 96.8%, 79.7% vs. 95.0%, 75.1% vs.93.9%, 73.2% vs. 93.6% and 73.2% vs. 93.6% at 12, 24, 36, 48 and 60 months, respectively. The primary patencies of AVF in group B were 95.0%, 91.7%, 89.2%, 88.3% and 88.3% at 12, 24, 36, 48 and 60 months, respectively. After adjusting for potential confounders, age, angiotensin-converting inhibitors or angiotensin-receptor blockers (anti-RAAS) drugs and D-dimer were independent predictors of AVF failure. However, there were no differences between functional and failed AVF regarding all-cause mortality.

**Conclusions**: The study revealed that the primary and secondary patiencies of the surgery of new AVF proximal to the failed ones were ideal operations to restore failed forearm AVF.

## Introduction

Autologous arteriovenous fistula (AVF) is the preffered access for most patients receiving maintenance hemodialysis (HD), and associated with lower mortality and lower infection rates compared with the other two modalities of vascular access (central venous catheters and grafts) used for chronic HD [1,2]. However, failed AVF is a major issue in the creation of functional hemodialysis vascular access. In fact, there are large international differences exist in AVF location, successful use of AVF, and time to first AVF use, challenging what constitutes best practice [3]. A recent systematic review and meta-analysis demonstrated that for fistulas, the primary and secondary patencies at one year were only 64% and 79%, respectively, and 21% of fistulas were abandoned without use [4]. In clinical practice, there is substantial international variation in the use of AVF, as successful AVF use was 87% in Japan, 67% in Europe, and only 64% in the United States [3].

In fact, thrombosis and stenosis are the main causes of AVF failure, and the anatomic abnormalities of AVF caused by stenosis contributes to the enhancement of thrombosis [5]. These clinical practices have considered access procedures to include endovascular interventions such as angioplasty, thrombolysis, thrombectomy, or surgical revisions. Both surgical and percutaneous transluminal angioplasty (PTA) techniques are at the disposal of the physician involved in AVF management. Recent studies evaluating long-term outcomes in AVF requiring interventions to promote maturation report primary patency rates ranging from 28% to 72% and secondary patency rates of 68% to 96% at 1 year [6]. It is unclear whether one method is preferred over the other and which short- and long-term outcomes can be expected [7–9]. Recent published studies demonstrated that the surgery of new AVF proximal to the failed ones had superior survival times over PTA for the treatment of failed AVF [6,10,11]. The possible mechanism is that the vessel injury resulting from PTA is usually greater than that obtained by surgical revision [12]. However, most of these studies lacked long-term follow-up, and lacked end point of the mortality [13]. Moreover, endovascular treatments for AVF dysfunction and failure were limited in past few years in China because of the laggard equipment and techniques,and especially lacking of health insurance coverage. The surgery of new AVF proximal to the failed ones is the traditional treatment for restoring failed AVF and is also inexpensive in China. Therefore, dialysis units should evaluate an appropriate standard of care to reduce the AVF failure rate and to improve the successful rate of restored AVF [14]. Accordingly, we conducted a retrospective cohort study to evaluate the clinical outcomes of the surgery of new AVF proximal to the failed forearm AVF.

## Material and methods

### Participants and study design

**Figure 1.**
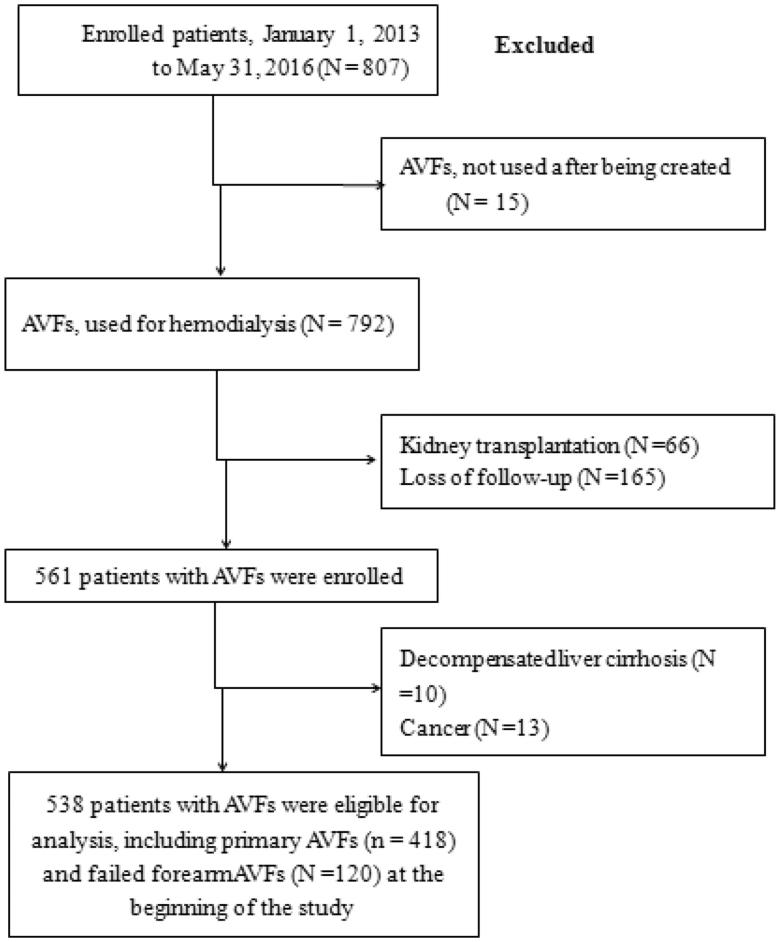
Study cohort derivation.

In this single-center retrospective cohort study, 1501 ESRD patients were treated and consecutively enrolled in the Department of Nephrology, Shandong Provincial Qianfoshan Hospital, which is affiliated with Shandong University, between January 2013 and June 2016. The study focused on functional (able to provide adequate dialysis delivery) and mature AVFs created at the cephalic vein to radial artery, of the left or right lower half of the forearm. Secondary patency data were available during follow-up. The deadline of follow-up was November 10^th^, 2018. All patient data were stored in the electronic database YIDUCLOUD (http://192.168.160.2). The exclusion criteria depicted in Figure 1 were as follows: (1) age < 18 years; (2) initiated HD therapy following a failed kidney transplantation; (3) transitioned to peritoneal dialysis therapy within 6 months of HD initiation; (4) presence of malignant disease, hypotension, active bleeding, or severe liver disease; (5) received local or peripheral venous thrombolytic therapy solely after AVF thrombosis; (6) received the PTA treatment; and (7) no known AVF patency at the end of the follow-up period. After excluding patients meeting these criteria, a total of 807 patients were enrolled. Additionally, 269 patients (15 patients with AVFs not used after being created; 66 patients received kidney transplantation during the follow-up period; 165 patients lost to follow-up; 10 patients diagnosed with decompensated liver cirrhosis and 13 patients diagnosed with cancer) were also excluded. Therefore, a total of 538 patients were eligible for analysis in the study. Of these patients, 418 patients received a primary AVF (group A), while 120 patients, with a failed forearm AVF, underwent proximal neo-anastomosis or conversion to upper arm AVF (group B). The median cohort follow-up was 41 months. All patients used 16 G needles for dialysis and were treated with low molecular weight heparin anticoagulation only on the day of dialysis. The anticoagulant dose was 40–60 IU/kg intravenously.

### Technical notes

The surgery of new AVF proximal to the failed forearm AVF was performed as an inpatient procedure under local anesthesia and consisted of either the creation of a more proximal reanastomosis of the cephalic vein to the radial artery or to the brachial artery, or the basilica vein to the brachial artery. An end-to-side anastomosis was created in the standard fashion. Technical success including: (1) improved intra-access blood flow > 20%; (2) normalized venous pressures; (3) normalized prepump pressures and return to baseline blood flow rate and (4) Abnormal duplex ultrasound return to baseline duplex ultrasound Doppler flows > 4–500 mL/min [15]. The procedure was considered anatomically successful when AVF was effectively used for HD. Successful AVF use was defined as a newly created AVF used for 30 or more continuous days for typically thrice-weekly HD [15,16].

### Data collection, definitions, and outcomes

Demographic characteristics (age, sex, smoking status and use of prescription drugs), primary renal diseases, comorbidities (hypertension, diabetes and cardiovascular disease), and history of AVF creation at baseline were recorded. Blood was collected by means of venupuncture after an overnight fast of at least 10 h. Laboratory findings, including hemoglobin, calcium, phosphorus, magnesium, parathyroid hormone (PTH), urea nitrogen, serum creatinine, fasting blood glucose, serum uric acid, serum total cholesterol (TC), and triglycerides (TG), were collected on the day before AVFs surgery, which were measured by the automatic biochemistry analyzer in the central laboratory of Qianfoshan Hospital. Diabetes was defined as fasting blood glucose ≥ 7.0 mmol/L or by the use of hypoglycemic agents or by a self-reported history of diabetes. Hypertension was defined as a systolic blood pressure of more than 140 mm Hg and/or diastolic blood pressure of more than 90 mm Hg, or current prescription use of antihypertensive drugs. Cardiovascular disease (CVD) included ischemic heart disease, heart failure, stroke and peripheral vascular disease.

### Failed AVF

Failed AVF was defined as its failing to mature at 12 weeks post surgery, or the presence of stenosis more than 50% or thrombosis, and meanwhile lost the ability to receive prescribed hemodialysis [16,17]. National Dialysis Registration System and YIDUCLOUD data were linked using a patient identifier, allowing us to determine the first month in which the AVF was being used for hemodialysis (defined as successful 2-needle cannulation) subsequent to the AVF placement date, which reflects clinical AVF maturation. The outflow veins were assessed for sclerosis, stenosis, thrombosis, patency, and continuity by duplex ultrasound imaging.

### Outcomes

Clinical follow-up was undertaken in the dialysis unit where details of success or complications with AVF use were recorded. Patients were also contacted by telephone to incorporate a structured interview that included questions on frequency and success at HD, complications (such as stenosis and thrombosis), and any subsequent treatment [18]. End points were failure and restoration by the surgery of new AVF proximal to the failed ones. The primary patency was defined as the time of access creation or placement until any first intervention to maintain or restore blood flow, first occurrence to access thrombosis, or reaching a censored event; The secondary patency was defined as the interval from AVF placement until AVF abandonment, or the time of patency measurement including restoration by the surgery of new AVF proximal to the failed ones designed to re-establish functionality in the failed AVF [15,19]. End points also included death from any cause.

### Statistical analyses

Data are presented as proportions for categorical variables and mean ± SD or median [interquartile range (IQR)] for continuous variables. The significance of differences in continuous variables between groups was analyzed using a t test or Wilcoxon rank-sum test of variables. The difference in the distribution of categorical variables was analyzed using the Chi-square test. Primary and secondary patency rates of AVF were evaluated using the Kaplan Meier method, and the log-rank test was used for differences between the groups. The cumulative patency rates were calculated for 12, 24, 36, 48, and 60 months. During the follow-up period in group A, 1 patient committed suicide, 2 patients died due to traffic accidents, 15 patients were lost to follow-up; 4 patients were lost to follow-up in group B. Next, we confirmed the proportional hazard assumption and conducted Cox proportional hazards analyses for risk factors associated with the failure of AVF. Baseline variables including age, sex, smoking status, hypertension, diabetes, CVD, angiotensin-converting inhibitors or angiotensin-receptor blockers (anti-RAAS) drugs, statins, antiplatelet drugs, hemodialysis, hemoglobin, calcium, phosphorus, PTH, serum albumin, triglycerides, total cholesterol, uric acid, creatinine and D-dimer that were considered clinically relevant were entered into the multivariable regression models. Crude and adjusted hazard ratios (HRs) with 95% confidence intervals (CIs) were reported. Furthermore, we used the Kaplan–Meier survival curve analysis to explore the association between the functional status of AVF and all-cause mortality.

All analyses were performed with SPSS statistical package, version 16.0 (SPSS, Inc., Chicago, IL). All *p*-values are two tailed. A *p* value of less than .05 was considered statistically significant.

### Ethics approval and consent to participate

The study was conducted in accordance with the Declaration of Helsinki, and also under the approval of the Ethics Committee of Qianfoshan Hospital, Shandong University (2019S015). The informed consents from patients in the study were waived, however, patients are informed about the registration of all individuals with treated ESRD by the nephrology clinic as well as about their right to not participate in the study.

## Results

### Baseline characteristics

The baseline characteristics of the participants stratified according to the AVF functionality status (group A and group B) at the time of inclusion are shown in Table 1. The primary cause of ESRD was chronic glomerulonephritis (39.0% in group A and 56.7% in group B). For group A, the mean age was 54.5 ± 14.5 years (range from 18 to 87 years), and 59.8% of the patients were male. For group B, the mean age was 50.9 ± 14.7 years (range from 20 to 85 years), and 65.0% of the patients were male. In group A, male patients had higher levels of hemoglobin, serum albumin, creatinine, uric acid and also had higher percentage of diabetes than females, respectively (*p* < .05). In group B, male patients also had higher levels of hemoglobin, creatinine and uric acid than females, respectively (*p* < .05), however, the percentage of diabetes was not different between males and females. There were 113 patients (27.0%) with AVF failure in group A and 15 patients (12.5%) with AVF re-failure in group B during the follow-up period.

**Table 1.** Baseline characteristics of participants stratified according to the status of AVFs.

	Group A, primary AVFs (*n* = 418)			Group B, failed forearm AVFs (*n* = 120)		
	Male (*n* = 250)	Female (*n* = 168)	*p* value	Male (*n* = 78)	Female (*n* = 42)	*p* value
Age (years)	53.6 ± 14.0	55.7 ± 15.2	.15	50.0 ± 14.7	52.4 ± 14.7	.39
Follow-up time (month, IQR)	41.0 (31.0–50.5)	40.0 (30.0–50.0)	.48	41.0 (30.5–54.0)	40.0 (17.0–49.0)	.27
Causes (*n*, %)			**.002**			.59
CGN	86 (34.4)	77 (45.8)		47 (60.3)	21 (50.0)	
DKD	95 (38.0)	41 (24.4)		10 (12.8)	9 (21.4)	
HTN	32 (12.8)	10 (6.0)		6 (7.7)	6 (14.3)	
Others	37 (14.4)	40 (23.8)		15 (19.2)	6 (14.3)	
Smoking (*n*, %)	118 (47.2)	3 (1.8)	**< .001**	27 (34.6)	1 (2.4)	**< .01**
Hypertension (*n*, %)	226 (90.4)	144 (85.7)	.16	66 (84.6)	37 (88.1)	.79
Diabetes (*n*, %)	102 (40.8)	44 (26.2)	**.002**	10 (12.8)	7 (16.7)	.59
CVD (*n*, %)	90 (36.0)	51 (30.4)	.25	20 (25.6)	11 (26.2)	1.0
Receiving HD (*n*, %)	182 (72.8)	104 (61.9)	**.03**	75 (96.2)	41 (97.6)	1.0
Hemoglobin (g/L)	88.1 ± 20.9	83.4 ± 21.3	**.03**	111.5 ± 21.3	100.5 ± 20.3	**< .01**
Serum albumin (g/L)	34.1 ± 6.2	35.3 ± 6.5	.05	40.0 ± 5.7	38.3 ± 6.9	.16
Calcium (mmol/L)	1.99 ± 0.27	2.05 ± 0.27	.05	2.20 ± 0.30	2.15 ± 0.27	.41
Phosphorus (mmol/L, IQR)	1.84 (1.48–2.20)	1.75 (1.41–2.17)	.3	2.06 (1.69–2.56)	1.85 (1.52–2.28)	.11
Serum Magnesium (mmol/L)	1.03 ± 0.21	1.07 ± 0.21	.1	1.16 ± 0.20	1.18 ± 0.24	.73
iPTH (pg/mL)	227.4 (139.4–339.2)	218.3 (104.0–417.5)	.93	274.0 (106.4–506.8)	291.5 (138.8–654.5)	.22
Creatinine (mg/dL, IQR)	8.0 (5.8–11.3)	7.3 (5.6–9.6)	**.03**	11.0 (8.8–13.9)	9.4 (6.6–11.3)	**.01**
Uric acid (mg/dL, IQR)	7.5 (5.9–9.1)	7.0 (5.2–8.5)	**.04**	6.9 (5.7–8.5)	5.9 (4.6–6.9)	**< .01**
Triglycerides (mmol/L, IQR)	1.30 (0.88–1.83)	1.50 (0.97–2.05)	.09	1.11 (0.78–2.0)	1.73 (1.13–2.19)	**.03**
Total cholesterol (mmol/L, IQR)	4.29 (3.56–4.99)	4.77 (3.94–5.66)	**< .001**	3.70 (3.02–4.38)	4.59 (3.84–5.38)	**< .01**
D-dimer (mg/L, IQR)	1.21 (0.55–2.47)	1.17 (0.68–2.54)	.73	0.73 (0.26–1.40)	1.11 (0.35–2.13)	.17

AVF: autologous arteriovenous fistula; CGN: chronic glomerulonephritis; DKD: diabetic kidney disease; HTN: hypertensive nephrology; CVD: cardiovascular disease; HD: hemodialysis; PTH: parathyroid hormone.

The significance of bold values represents the *p* values less than .05.

### Longer-term outcomes of AVFs

**Figure 2.**
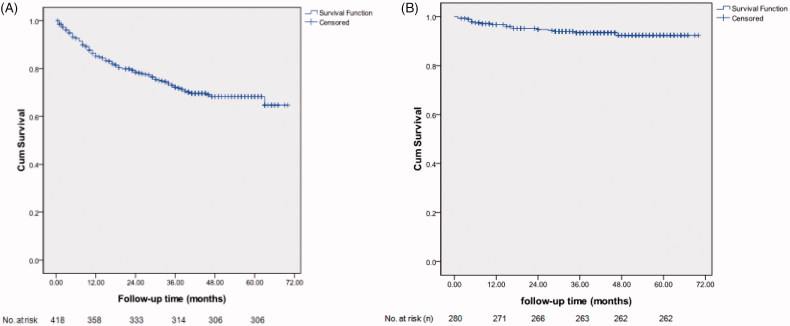
Kaplan–Meier survival curve analysis of primary (A) and secondary (B) patencies of primary AVFs in group A.

**Figure 3.**
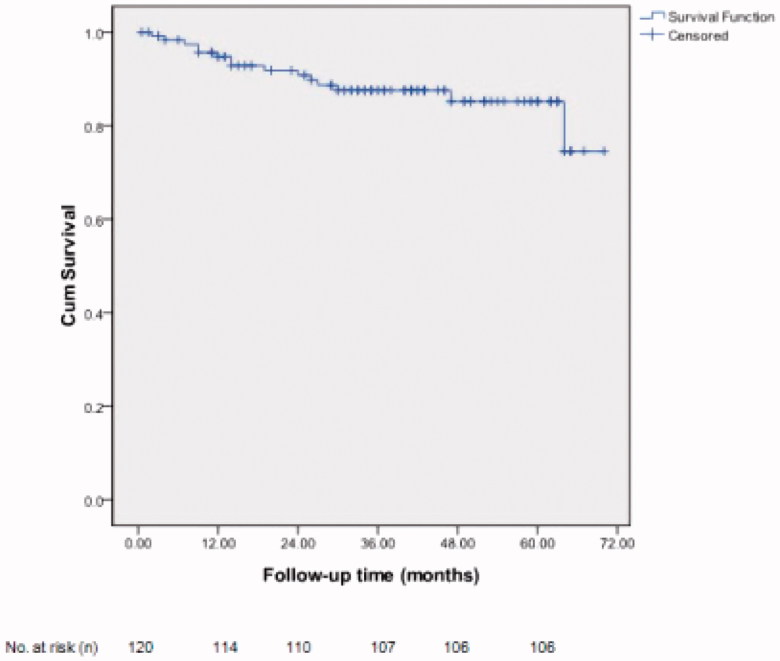
Kaplan–Meier survival curve analysis of patency of restoration of failed AVFs in group B (*N* = 120).

In group A (*n* = 418), the primary patency of AVF was 85.6%, 79.7%, 75.1%, 73.2% and 73.2% at 12, 24, 36, 48 and 60 months, respectively. Most failed AVFs in group A were restored by the surgery of new AVF proximal to the failed ones. After the restoration, the secondary patencies were 96.8%, 95.0%, 93.9%, 93.6% and 93.6% at 12, 24 36, 48, and 60 months, respectively, Figure 2. In group B (*n* = 120), the primary patencies of restoration by the surgery of new AVF proximal to the failed forearm AVF was 95.0%, 91.7%, 89.2%, 88.3% and 88.3% at 12, 24, 36, 48 and 60 months, respectively, Figure 3.

Males had better patency rates than females both in group A and in group B. Compared with older patients (age ≥65years), younger patients (<65years) had better patency rates in group A, however, t younger patients did not have better patency than older patients in group B (Supplementary Figure 1 and Supplementary Figure 2).

### Factors associated with AVF failure

We confirmed the proportional hazard assumption and conducted Cox proportional hazards regression analyses for risk factors associated with AVF failure (group A). In the crude Cox regression analysis, age (per 10 years’ increase), anti-RAAS drugs, statins, antiplatelet drugs and D-dimer (per 1 mg/L increase) were independently associated with AVF failure, with HRs of 1.11 (95% CI, 1.04 to 1.20), 1.56 (95% CI, 1.26 to 1.94), 1.77 (95% CI, 1.30 to 2.40), 1.48 (95% CI, 1.12 to 1.96) and 1.07 (95% CI, 1.03 to 1.11), respectively. After adjusting for potential confounders, age (HR 1.19, 95% CI, 1.05–1.35), anti-RAAS drugs (HR 1.61, 95% CI, 1.15–2.25) and D-dimer (HR 1.07, 95% CI, 1.02–1.12) were independent predictors of AVF failure (Table 2).

**Table 2.** Cox proportional hazards analyses for risk factors associated with failure of primary AVFs in group A (*n* = 418).

Variables	Crude HR (95% CI)	Age- and sex-adjusted HR^a^ (95% CI)	Multivariable adjusted HR^b^ (95% CI)
Age (Per 10 years increase)	**1.11 (1.04–1.20)**	**1.11 (1.03–1.19)**	**1.19 (1.05–1.35)**
Gender (females vs. males)	**1.23 (1.01–1.50)**	1.18 (0.97–1.44)	0.87 (0.60–1.27)
Smoking (yes vs. no)	0.91 (0.73–1.12)	1.0 (0.78–1.28)	0.88 (0.60–1.29)
Hypertension (yes vs. no)	1.15 (0.85–1.56)	1.16 (0.86–1.58)	1.24 (0.73–2.10)
Diabetes (yes vs. no)	1.08 (0.89–1.33)	1.02 (0.82–1.26)	0.78 (0.55–1.12)
CVD (yes vs. no)	1.08 (0.88–1.32)	1.01 (0.81–1.25)	0.88 (0.61–1.28)
Anti-RAAS drugs (yes vs. no)	**1.56 (1.26–1.94)**	**1.55 (1.25–1.93)**	**1.61 (1.15–2.25)**
Statins (yes vs. no)	**1.77 (1.30–2.40)**	**1.70 (1.24–2.32)**	1.16 (0.65–2.09)
Antiplatelet drugs (yes vs. no)	**1.48 (1.12–1.96)**	**1.43 (1.07–1.90)**	1.40 (0.81–2.44)
Hemodialysis (yes vs. no)	0.91 (0.74–1.12)	0.99 (0.80–1.22)	0.95 (0.64–1.39)
Hemoglobin (per 10g/L increase)	1.01 (0.97–1.06)	1.01 (0.97–1.06)	0.97 (0.89–1.05)
Calcium (per 1mmol/L increase)	1.09 (0.78–1.54)	1.07 (0.75–1.51)	0.98 (0.48–2.02)
Phosphorus (per 1mmol/L increase)	0.95 (0.85–1.08)	0.99 (0.88–1.12)	0.97 (0.75–1.25)
PTH (per 100pg/mL increase)	0.99 (0.96–1.02)	1.0 (0.97–1.04)	1.02 (0.96–1.09)
Serum albumin (per 10g/L increase)	1.04 (0.88–1.22)	1.04 (0.89–1.22)	1.12 (0.83–1.50)
Triglycerides (per 1mmol/L increase)	1.02 (0.91–1.14)	1.02 (0.91–1.14)	0.97 (0.81–1.16)
Total cholesterol (per 1mmol/L increase)	1.04 (0.96–1.12)	1.03 (0.96–1.11)	1.04 (0.92–1.17)
Uric acid (per 1mg/dL increase)	0.98 (0.95–1.02)	0.98 (0.95–1.02)	1.06 (0.98–1.15)
Creatinine (per 1mg/dL increase)	0.97 (0.95–1.0)	0.98 (0.96–1.01)	0.96 (0.90–1.02)
D-dimer (per 1 mg/L increase)	**1.07 (1.03–1.11)**	**1.07 (1.03–1.11)**	**1.07 (1.02–1.12)**

AVF: autologous arteriovenous fistula; HR: hazard ratio; CI: confidence interval; CVD: cardiovascular disease; Anti-RAAS drugs: including angiotensin-converting inhibitors and angiotensin-receptor blockers; PTH: parathyroid hormone.

The significance of bold values represents the *p* values less than .05.

a HR was adjusted for age and sex.

b HR was adjusted for age, gender, smoking, hypertension, diabetes, CVD, anti-RAAS drugs, statins, antiplatelet drugs, hemodialysis, hemoglobin, calcium, phosphorus, PTH, serum albumin, triglycerides, total cholesterol, uric acid, creatinine, D-dimer.

### Association between functional status of AVF and all-cause mortality

**Figure 4.**
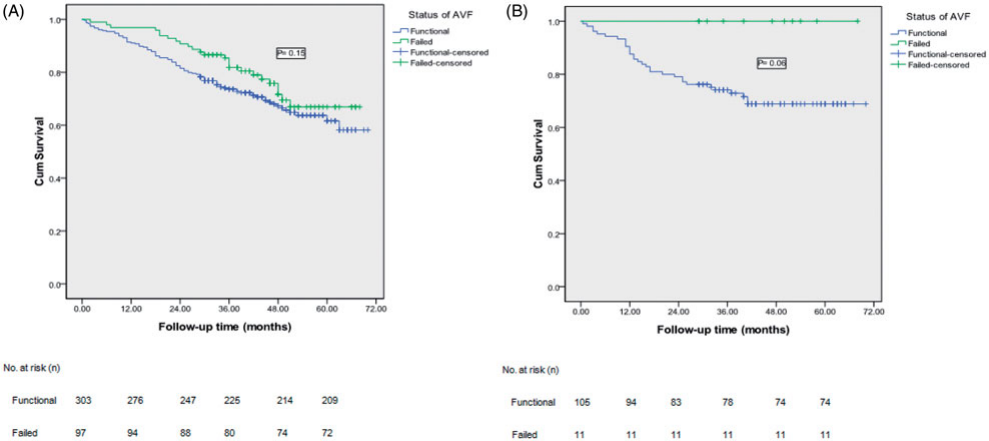
Kaplan–Meier survival curve analysis of mortality between functional and failed AVF groups. A, (Group A, *n* = 400, functional vs. failed, *p* = .07); B, (Group B, *n* = 116, functional vs. failed, *p* = .51).

Furthermore, we used the Kaplan–Meier survival curve analysis to explore the association between the functional status of AVF and all-cause mortality. During the follow-up period in group A, 1 patient committed suicide, 2 patients died due to traffic accidents, 15 patients were lost to follow-up; in total, 120 patients died and 280 patients remained alive. The survival rates were not different between patients with functional and failed AVFs in group A (*N* = 400), which were 91.1% vs. 96.9%, 81.5% vs. 90.7%, 74.3% vs. 82.5%, 70.6% vs. 76.3%, and 69.0% vs. 74.2% at 12, 24, 36, 48 and 60 months, respectively (*p* = .15), Figure 4. Moreover, during the follow-up period in group B, 4 patients were lost to follow-up; in total, 31 patients died and 85 patients remained alive. The results also demonstrated no difference between functional and re-failed AVF patients in group B (*N* = 116), which were 89.5% vs. 100%, 79.0% vs. 100%, 74.3% vs. 100%, 78.7% vs. 100%, and 78.7% vs. 100% at 12, 24, 36, 48 and 60 months, respectively (*p* = .06), Figure 4.

## Discussion

Successful establishment of an optimally functioning AVF is a highly desirable result that can directly improve patient outcomes and lower the cost of care. Vascular access outcomes in hemodialysis are critically important for patients and clinicians. The international Standardized Outcomes in Nephrology (SONG) initiative proposed outcomes measures for function included ‘uninterrupted use of the access without the need for interventions’ and ‘ability to receive prescribed dialysis’, but not ‘access blood flow’ [13]. Our study demonstrated that the primary patency at 1 year were 85.6%, which was much better than in the United States [17]. In fact, there are large international differences in the location and use of AVF created for hemodialysis. From Dialysis Outcomes and Practice Patterns Study (DOPPS) 1 to 5, the percentage of AVF created in the lower arm was consistently ≥ 93% in Japan, but this value declined from 70% (DOPPS 1) to 32% (DOPPS 5) in the United States; Successful AVF use was 87% in Japan, and only 64% in the United States [3]. The potential negative aspects of upper-arm AVFs with a higher frequency of steal syndrome [20] and potential adverse long-term effects of high AVF-associated high blood flow on cardiac function should also be considered [21]. In our dialysis unit, AVF placement in distal upper-extremity sites was the first choice for AVF creation when feasible, and successful AVF use was approximately 79% at 1 year [18]. We do not know all the reasons that our patients attain such high levels of successful AVF use. Both dialysis and practitioner practices might have effects on the AVF survival. A much lower median blood flow rate was used in our patients (about 200 mL/min) than in the United States(about 425 mL/min) [3]. Asano et al. [22] found lower facility median blood flow rates to be associated with longer cumulative functional AVF survival. However, failure of AVFs is a complex pathophysiological process in which multiple factors interact. But ultrasound confirmed stenosis was present in 64% of well-functioning mature AVFs [23]. For example in some patients with the expansion of aneurysm, although the stenosis was more than 50%, the fistula might work well. Whether the imaging abnormalities without symptoms need to be dealt with is still controversy. This retrospective study was conducted to explore the longer-term outcomes of restorative surgery of failed AVFs. A recent meta-analysis of the efficacy and outcomes of AVF for HD showed that for fistulas, the primary unassisted and secondary patency rates at 1 year were 64% and 79%, respectively [4]. This study demonstrated that the primary and secondary patencies at 1 year were 85.6% and 96.8%, 79.7% and 95.0% at 2 years, 73.2% and 93.6% at 5 years in group A; the primary patencies of AVF in group B were 95.0%, 91.7%, and 88.3% at 1, 2, and 5 years, which means that the patencies were ideal after the surgery of new AVF proximal to the failed ones. Hence, special attention should be given to the possibility that the potential clinical utility of the surgery of new AVF proximal to the failed ones may be suitable when long-term follow-up is considered.

Although an AVF is considered the preferred type of access, recent studies demonstrate that about 20–60% of AVFs fail to mature for successful dialysis use [24,25], which further confirms the importance of intensive examinations for the early detection of clarifying risk factors to prevent the occurrence of AVF failure. This study demonstrated that males had better patency than females, which may be due to larger arterial diameter in males [26]. Moreover, this study demonstrated that elderly patients had worse patency of AVF than younger patients in group A, probably due to higher vascular stiffness caused by numerous comorbidities, such as hypertension, diabetes and cardiovascular disease. Some of these diseases, such as severe arteriopathy are classic predictors of AVF failure [27,28]. However, there was no difference in patency between elderly and younger patients after restoration by the surgery of new AVF proximal to the failed forearm AVF in group B. It is not surprising that those with previous AVF may have good usable veins, and a higher rate of successful restoration of failed AVF. Notably, our study revealed that, after adjusting for confounders, the use of anti-RAAS drugs was an independent predictor of AVF failure (HR 1.61, 95% CI, 1.15–2.25). One possible reason is that the occurrence of hypotension is higher, and also, patients prescribed with anti-RAAS drugs may also have severe CVD. However, this conclusion is merely speculative and lacks supporting data that are needed from further studies to study this explanation. In this study, we also found D-dimer to be a risk factor for AVF failure, which is related to thrombosis. It is known that thrombosis is an important risk factor for AVF failure. Previous studies revealed that PTA might cause injury to the vascular endothelium and therefore trigger proliferative repair, causing recurrent thrombosis that leads to the AVF failure [29,30]. Recent studies demonstrated that the surgery of new AVF proximal to the failed ones had superiorities in survival time than PTA for the treatment of failed AVFs [6,10,11], which may be related to the possible mechanism that the vessel injury resulting from PTA is usually greater than that caused by surgical revision [12].

AVF function is highly relevant, important, and appropriate as the core outcome for AVF success because its applicability can impact the quality of life and survival of patients. However, most of current studies show that a lack of long-term follow-up and a core outcome of failed AVF intervention affect mortality [13]. This study demonstrated that the survival rates were not different between functional and failed AVFs in both group A and group B.

This study also has limitations that deserve attention. First, this is a single-center study, and our prediction models are not calibrated to any other centers, therefore, selection bias in the study limited the extension of the results from this study to other populations. Second, there was a lack of ultrasound findings, such as the presence of vascular calcification and postoperative venous dilatation, which provide important prognostic information, as seen in some reports [27,31]. Third, This study have relatively high percentage of censoring events, which might result in overestimating the patencies of AVF. Finally, despite adjustment for multiple variables in the Cox regression analysis, we could not rule out other confounders that need to be explored in future studies.

## Conclusion

In summary, this study revealed that the primary and secondary patiencies of the surgery of new AVF proximal to the failed ones were ideal operations to restore failed forearm AVF. Considering hemodialysis patients lived longer for the advancements in new drug treatment, equipment and techniques. Therefore, from the opinion of the protection of vascular access resource, if patients can obtain the most benefits from the surgery of new AVF proximal to the failed ones need further research. Center experience is considered of major importance in choosing an appropriate standard of care to reduce the AVF failure rate and to improve the successful rate of restored AVF [14]. Further appropriately powered and randomized studies are still needed to clarifying this important issue. Moreover, we need to further confirm the importance of clarifying risk factors and conducting interventions to prevent the occurrence of AVF failure, improve the lifespan of AVF, and improve the quality of life of patients.

## Supplementary Material

Supplemental Material

## References

[1] Ravani P, Palmer SC, Oliver MJ, et al. Associations between hemodialysis access type and clinical outcomes: a systematic review. J Am Soc Nephrol. 2013;24(3):465–473. 23431075 10.1681/ASN.2012070643 PMC3582202

[2] Ravani P, Gillespie BW, Quinn RR, et al. Temporal risk profile for infectious and noninfectious complications of hemodialysis access. J Am Soc Nephrol. 2013;24(10):1668–1677. 23847278 10.1681/ASN.2012121234 PMC3785277

[3] Pisoni RL, Zepel L, Fluck R, et al. International differences in the location and use of arteriovenous accesses created for hemodialysis: results from the Dialysis Outcomes and Practice Patterns Study (DOPPS). Am J Kidney Dis. 2018;71(4):469–478. 29198387 10.1053/j.ajkd.2017.09.012

[4] Bylsma LC, Gage SM, Reichert H, et al. Arteriovenous fistulae for haemodialysis: a systematic review and meta-analysis of efficacy and safety outcomes. Eur J Vasc Endovasc Surg. 2017;54(4):513–522. 28843984 10.1016/j.ejvs.2017.06.024

[5] Gameiro J, Ibeas J Factors affecting arteriovenous fistula dysfunction: a narrative review. J Vasc Access. 2019:1129729819845562. 10.1177/1129729819845562 31113281

[6] Tordoir JHM, Zonnebeld N, van Loon MM, et al. Surgical and endovascular intervention for dialysis access maturation failure during and after arteriovenous fistula surgery: review of the evidence. Eur J Vasc Endovasc Surg. 2018;55(2):240–248. 29307757 10.1016/j.ejvs.2017.12.001

[7] Klimach SG, Norris JM Surgical versus endovascular management of thrombosed autogenous arteriovenous fistulae. Int J Surg. 2014;12(3):237–240. 24429061 10.1016/j.ijsu.2013.12.017

[8] Kuhan G, Antoniou GA, Nikam M, et al. A meta-analysis of randomized trials comparing surgery versus endovascular therapy for thrombosed arteriovenous fistulas and grafts in hemodialysis. Cardiovasc Intervent Radiol. 2013;36(3):699–705. 23381773 10.1007/s00270-013-0564-8

[9] Tordoir JH, Bode AS, Peppelenbosch N, et al. Surgical or endovascular repair of thrombosed dialysis vascular access: is there any evidence? J Vasc Surg. 2009;50(4):953–956. 19786244 10.1016/j.jvs.2009.06.058

[10] Tessitore N, Mansueto G, Lipari G, et al. Endovascular versus surgical preemptive repair of forearm arteriovenous fistula juxta-anastomotic stenosis: analysis of data collected prospectively from 1999 to 2004. Clin J Am Soc Nephrol. 2006;1(3):448–454. 17699244 10.2215/CJN.01351005

[11] Fan SS, Chen CW, Lu KC, et al. A comparison of efficacy of endovascular versus surgical repair for the treatment of arteriovenous fistula stenosis in Taiwan. J Vasc Access. 2017;18(3):200–206. 28218365 10.5301/jva.5000669

[12] Lee T, Tindni A, Roy-Chaudhury P Improved cumulative survival in fistulas requiring surgical interventions to promote fistula maturation compared with endovascular interventions. Semin Dial. 2013;26(1):85–89. 22404567 10.1111/j.1525-139X.2012.01060.x PMC3965258

[13] Viecelli AK, Tong A, O'Lone E, et al Report of the Standardized Outcomes in Nephrology-Hemodialysis (SONG-HD) consensus workshop on establishing a core outcome measure for hemodialysis vascular access. Am J Kidney Dis. 2018;71(5):690–700. 29478866 10.1053/j.ajkd.2017.12.003

[14] Tan RY, Pang SC, Teh SP, et al. Outcomes of endovascular salvage of clotted arteriovenous access and predictors of patency after thrombectomy. J Vasc Surg. 2019: pii: S0741-5214(19)31804-X. 10.1016/j.jvs.2019.07.056 31492611

[15] Lee T, Mokrzycki M, Moist L, et al. North American vascular access C: standardized definitions for hemodialysis vascular access. Semin Dial. 2011;24(5):515–524. 21906166 10.1111/j.1525-139X.2011.00969.x PMC3999346

[16] Tong A, Manns B, Hemmelgarn B, et al. Establishing core outcome domains in hemodialysis: report of the Standardized Outcomes in Nephrology-Hemodialysis (SONG-HD) consensus workshop. Am J Kidney Dis. 2017;69(1):97–107. 27497527 10.1053/j.ajkd.2016.05.022 PMC5369351

[17] Woodside KJ, Bell S, Mukhopadhyay P, et al. Arteriovenous fistula maturation in prevalent hemodialysis patients in the United States: a national study. Am J Kidney Dis. 2018;71(6):793–801. 29429750 10.1053/j.ajkd.2017.11.020 PMC6551206

[18] Zhang Y, Kong X, Tang L, et al. Analysis of follow-up methods of vascular access and patient outcomes in hemodialysis at a tertiary care hospital in China. Ther Apher Dial. 2018;22(2):160–165. 29349919 10.1111/1744-9987.12646

[19] Sidawy AN, Gray R, Besarab A, et al. Recommended standards for reports dealing with arteriovenous hemodialysis accesses. J Vasc Surg. 2002;35(3):603–610. 11877717 10.1067/mva.2002.122025

[20] Yu SH, Cook PR, Canty TG, et al. Hemodialysis-related steal syndrome: predictive factors and response to treatment with the distal revascularization-interval ligation procedure. Ann Vasc Surg. 2008;22(2):210–214. 18346574 10.1016/j.avsg.2007.12.005

[21] Wakabayashi K, Io H, Nakata J, et al. Effects of cardiac function with postoperative arteriovenous fistula blood flow in patients with hemodialysis. Blood Purif. 2017;44(1):24–29. 28237983 10.1159/000458146

[22] Asano M, Thumma J, Oguchi K, et al. Vascular access care and treatment practices associated with outcomes of arteriovenous fistula: international comparisons from the Dialysis Outcomes and Practice Patterns Study. Nephron Clin Pract. 2013;124(1–2):23–30. 24029920 10.1159/000353733

[23] Pietura R, Janczarek M, Zaluska W, et al. Colour Doppler ultrasound assessment of well-functioning mature arteriovenous fistulas for haemodialysis access. Eur J Radiol. 2005;55(1):113–119. 15950108 10.1016/j.ejrad.2004.09.010

[24] Schinstock CA, Albright RC, Williams AW, et al. Outcomes of arteriovenous fistula creation after the Fistula First Initiative. Clin J Am Soc Nephrol. 2011;6(8):1996–2002. 21737851 10.2215/CJN.11251210 PMC3156429

[25] Huijbregts HJ, Bots ML, Wittens CH, CIMINO study group, et al. Hemodialysis arteriovenous fistula patency revisited: results of a prospective, multicenter initiative. Clin J Am Soc Nephrol. 2008;3(3):714–719. 18256379 10.2215/CJN.02950707 PMC2386712

[26] Joseph Lo Z, Tay WM, Lee Q, et al. Predictors of radio-cephalic arteriovenous fistulae patency in an Asian population. J Vasc Access. 2016;17(5):411–416. 27516144 10.5301/jva.5000591

[27] Jankovic A, Damjanovic T, Djuric Z, et al. Impact of vascular calcifications on arteriovenous fistula survival in hemodialysis patients: a five-year follow-up. Nephron. 2015;129(4):247–252. 10.1159/000380823 25823466

[28] Choi SJ, Yoon HE, Kim YS, et al. Pre-existing arterial micro-calcification predicts primary unassisted arteriovenous fistula failure in incident hemodialysis patients. Semin Dial. 2015;28(6):665–669. 25787294 10.1111/sdi.12365

[29] Bountouris I, Kristmundsson T, Dias N, et al. Is repeat PTA of a failing hemodialysis fistula durable? Int J Vasc Med. 2014;2014:1. 10.1155/2014/369687 PMC3920629 24587906

[30] Vesely TM, Siegel JB Use of the peripheral cutting balloon to treat hemodialysis-related stenoses. J Vasc Interv Radiol. 2005;16(12):1593–1603. 16371523 10.1097/01.RVI.0000190928.19701.DD

[31] Robbin ML, Greene T, Allon M, et al. Prediction of arteriovenous fistula clinical maturation from postoperative ultrasound measurements: findings from the hemodialysis fistula maturation study. J Am Soc Nephrol. 2018;29(11):2735–2744. 30309898 10.1681/ASN.2017111225 PMC6218859

