# Supplementary material for: Clinical outcomes following the surgery of new autologous arteriovenous fistulas proximal to the failed ones in end-stage renal disease patients: a retrospective cohort study
Source: Ren Fail. 2019 Dec 9;41(1):1036–44. doi: 10.1080/0886022X.2019.1696210 (PMC6913653; doi:10.1080/0886022X.2019.1696210)

**Supplementary fig 1.** Kaplan-Meier survival curve analysis of primary patencies of primary AVFs according to different sex and ages in group A. A, males vs. females, p = 0.03; B, <45years vs. 45-65years vs. ≥65years, p = 0.005.

A B


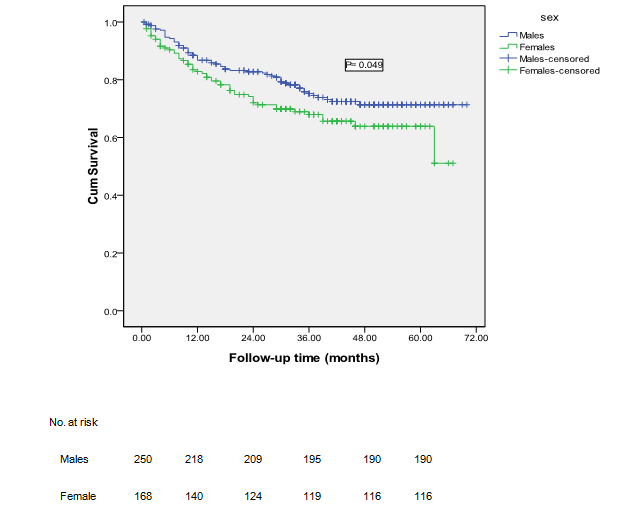

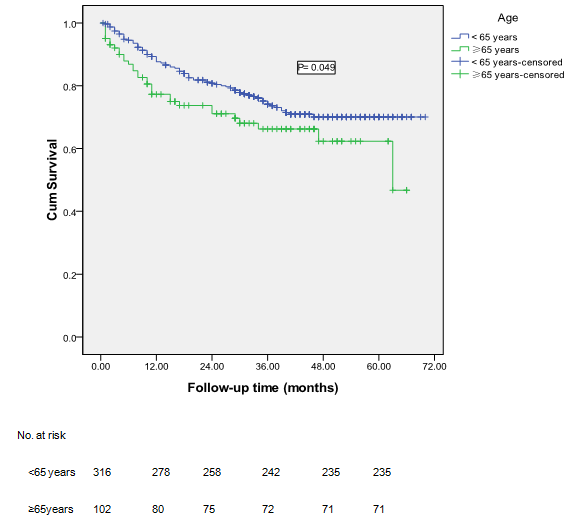


**Supplementary fig 2.** Kaplan-Meier survival curve analysis of patency of restoration of failed AVFs according to different sex and ages in group B; A, males vs. females, p = 0.29; B, <45years vs. 45-65years vs. ≥65years, p<0.001.

A B


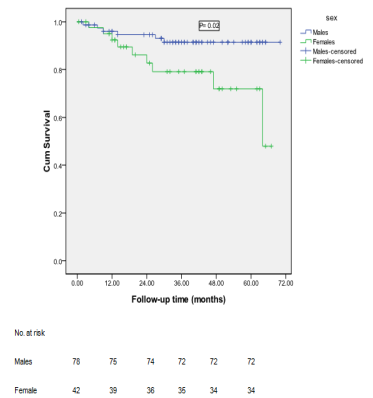

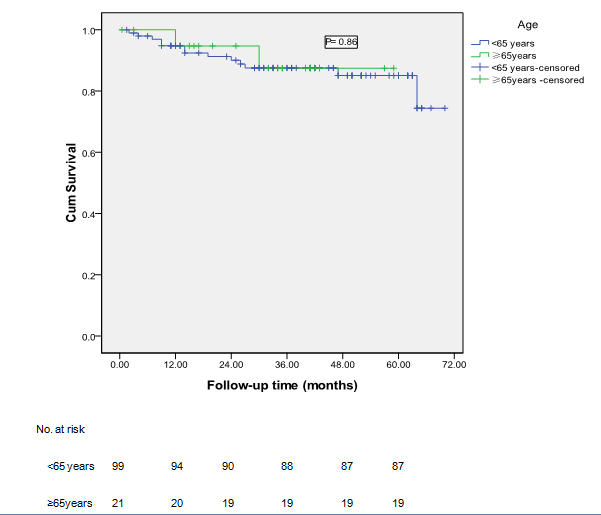

Supplement: Supplemental Material [file IRNF_A_1696210_SM9870.docx]
